# Quetiapine augmentation of SRIs in treatment refractory obsessive-compulsive disorder: a double-blind, randomised, placebo-controlled study [ISRCTN83050762]

**DOI:** 10.1186/1471-244X-5-5

**Published:** 2005-01-24

**Authors:** Paul D Carey, Bavanisha Vythilingum, Soraya Seedat, Jacqueline E Muller, Michael van Ameringen, Dan J Stein

**Affiliations:** 1 MRC Research Unit on Anxiety Disorders, University of Stellenbosch, Cape Town, South Africa; 2 Department of Psychiatry and Behavioral Neurosciences, McMaster University, Hamilton, Ontario, Canada

## Abstract

**Background:**

Although serotonin reuptake inhibitors are effective in the treatment of OCD, many patients fail to respond to these agents. Growing evidence from open-label and placebo-controlled trials suggests a role for augmentation of SRIs with atypical antipsychotics in OCD. Quetiapine is generally well tolerated and previous open-label data has produced mixed results in OCD and additional controlled data is needed.

**Methods:**

We undertook a double-blind, randomised, parallel-group, flexible-dose, placebo-controlled study of quetiapine augmentation in subjects who had responded inadequately to open-label treatment with an SRI for 12 weeks. Following informed consent and screening, forty-two subjects were randomised to either placebo or quetiapine for six weeks.

**Results:**

There was significant improvement from baseline to endpoint on the Yale-Brown Obsessive-Compulsive Scale in both the quetiapine and placebo groups (quetiapine, n = 20, p < 0.0001; placebo, n = 21, p = 0.001) with 40% (n = 8) of quetiapine and 47.6% (n = 10) of placebo treated subjects being classified as responders. Quetiapine did not demonstrate a significant benefit over placebo at the end of the six-week treatment period (p = .636). Similarly quetiapine failed to separate from placebo in the subgroup of subjects (n = 10) with co-morbid tics. Quetiapine was generally well tolerated.

**Conclusions:**

In this study, quetiapine augmentation was no more effective than placebo augmentation of SRIs. A number of limitations in study design make comparisons with previous studies in this area difficult and probably contributed to our negative findings. Future work in this important clinical area should address these limitations.

## Background

Obsessive-compulsive disorder (OCD) is a prevalent, chronic and disabling disorder [1]. Controlled pharmacotherapy studies have established superiority of serotonin re-uptake inhibitors (SRI's) over noradrenaline reuptake inhibitors and over placebo in OCD and these currently form the cornerstone of pharmacotherapy management [2]. Despite the considerable advances made with the introduction of the SRI's into clinical practice, 40–60% of subjects still fail to respond adequately to initial therapy [3,4].

From this it is clear that a need exists to pursue more effective treatments for those with OCD who fail to respond or respond inadequately to SRI's. To this end, preliminary evidence supports a role for the addition of atypical antipsychotics to SRIs in OCD. These agents combine serotonin-dopamine antagonism with the advantage of being well tolerated including a low potential for inducing motor side-effects.

To date a number of open-label studies have suggested that augmenting SRI's with atypical antipsychotics is an effective strategy for treatment-refractory OCD. These include support for risperidone [5-7], olanzapine [8-13], and more recently amisulpride [14] and quetiapine [15-18]. A single open-label study using quetiapine as augmentation showed lack of effect in a small sample using low doses [19].

The outcome of the first controlled study in this area with the antipsychotic haloperidol demonstrated preferential benefit for refractory OCD subjects with co-morbid tic disorder [20]. In two subsequent studies the efficacy of risperidone in SRI refractory OCD has also been reported [21,22]. Interestingly the former study [21], did not replicate the particular advantage for subjects with co-morbid tic disorder. Efficacy has also been shown for quetiapine [23] and olanzapine [24] using similar designs, but the effects on co-morbid tic disorders were not reported. In contrast a recent controlled study using olanzapine failed to demonstrate efficacy over placebo in a six week study [25].

Despite some mixed evidence in this area, in general the available literature appears to support the use of relatively short trials with low doses of antipsychotic agents as augmentation to SRIs. Quetiapine has a particularly interesting profile in that it is the only available antipsychotic with significant 5-HT1D effects and this serotonin receptor subtype has been implicated in OCD [26,27].

Our objective was to examine the effects of quetiapine augmentation in subjects with OCD who had failed to respond adequately to a 12 week trial of an SRI, employing a double-blind, placebo-controlled, six week study design.

## Methods

### Patients

Forty two subjects aged 18–65 years inclusive were recruited in our multi-centre study comprising five sites in South Africa and one in Canada. Recruitment took place between May 2002 and November 2003. Prior to commencement, all sites in the study received approval from their relevant Research Ethics committees/Institutional review boards and regulatory authorities. All subjects provided written informed consent prior to the commencement of any study-related procedures. Diagnosis was confirmed using the MINI Neuropsychiatric Interview (Version 5.00, 1998) [28] to ensure compatibility with the Diagnostic and Statistical Manual for Mental Disorders, Fourth Edition (DSM-IVTR)[29] criteria for OCD. Subjects with any co-existing Axis I disorder were excluded unless the co-morbid condition was deemed to be secondary to the OCD. Female subjects of childbearing potential were required to use adequate contraception and were not permitted to breastfeed while on the study. Subjects were excluded if they suffered from unstable medical conditions including renal or hepatic insufficiency, epilepsy or had suffered previous brain injury or undergone brain surgery. Taking medication that was deemed likely to interact with quetiapine or any other psychoactive substance was grounds for exclusion.

### Study Design

All subjects were treated and monitored by investigators for the minimum twelve week duration of SRI-alone treatment phase before inclusion into this study. This was to ensure that patients met criteria for duration of SRI treatment which included at least 6 weeks on the maximum tolerated dose of the relevant SRI. Sample size calculations were based conservatively on similar work in this area [20,21]. Accordingly, for the primary outcome variable (YBOCS), clinically meaningful differences between treatment groups of 6.67 with a standard deviation (SD) of 6 would be detected with a power of 80% at a 5% significance level with a sample size of 14 in each of the treatment groups. The larger sample recruited reflects the anticipation of a 33% drop-out rate in the double-blind treatment phase.

A double-blind, randomised, parallel-group six-week augmentation with quetiapine or matching placebo of the SRI to which participants had not responded adequately, was undertaken. Specific SRI's, mean doses and dose range are provided in Table 1. Non-responsiveness to an SRI was defined as either an improvement score on the clinical global impression scale of minimally improved (3) or worse (4,5,6), or less than 25% reduction in Yale Brown Obsessive-compulsive score following twelve weeks of treatment. Inadequate response, as defined above, to at least one SRI administered for a minimum of 12 weeks of which 6 weeks was either at the maximum tolerated dose or alternatively the manufacturer's recommended maximum daily dose. SRI doses were maintained at the same level throughout the double-blind treatment phase. For assignment to either quetiapine or placebo groups, we used a computer generated randomization schedule supplied by the sponsoring pharmaceutical company which also packaged the medication. This procedure ensured blinded, balanced allocation to each treatment group across all the study sites. All investigators remained blind to this schedule until closure of the study. No incidents requiring investigators to break the blind occurred through the course of the study.

**Table 1:** SRI's used by subjects for failed treatment trial prior to inclusion in the study.

**Drug**	**N**	**Mean maximum tolerated dose (mg/day)**	**Median daily dose (mg/day)**	**Range**
Fluoxetine	13	60	60	60
Citalopram	10	61	60	10
Paroxetine	4	65	60	20
Fluvoxamine	10	290	300	100
Sertraline	1	200	200	0
Clomipramine	2	250	250	0

### Treatment

At baseline participants were randomly allocated to receive treatment with either quetiapine or placebo using a computer generated schedule and numbered dispensing wallets. A flexible dosing schedule was initiated at 25 mg per day for one week and then doubled weekly to the start of week 4. Based on Clinical Global Impression of Improvement (CGI-I) scores of minimally improved or worse, clinicians were permitted to increase the dose to a maximum of 300 mg per day for the final two weeks of the study. In addition to clinical measures of improvement, clinicians also considered patient tolerability in their decision to adjust doses. Following completion of the treatment phase, subjects were withdrawn from study medication while continuing their SRIs. All subjects were then followed up for any adverse effects.

### Ratings

Patients were assessed by clinicians at baseline and on completion of weeks 2, 4 and 6. Telephonic assessments were performed on completion of weeks 1 and 3. Symptoms of obsessive-compulsive disorder were measured by the same clinician where possible at all study visits using the Yale Brown Obsessive Compulsive Scale YBOCS) [30,31]. A global assessment of severity and improvement was made by clinicians at all assessment points using the Clinical Global Impressions scale of Severity (CGI-S) and Improvement (CGI-I) [32]. Depression was rated using the 10-item Montgomery-Asberg Depression rating scale (MADRS) [33]. For a measure of patient-rated disability we used the Sheehan Disability scale (SDS) [34]. For subjects with tics, frequency and severity were rated using the Yale Global Tic Severity Scale (YGTSS) [35].

Our primary outcome measures for OCD symptoms were (1) the change in YBOCS score from baseline to endpoint and (2) the clinical global impression of improvement (CGI-I) at endpoint. In the final analysis, treatment response was defined as a 25% or greater reduction in YBOCS score and a CGI-I of 1 (very much improved) or 2 (much improved) from baseline to endpoint. Secondary outcome measures included the MADRS, SDS and YGTSS (in subjects with co-morbid tics).

### Statistical analysis

Thirty-nine of the forty-two randomised subjects successfully completed the six-week treatment phase. Two subjects withdrew from the study prematurely (Week 1 and Week 4) due to severe levels of sedation. In both of these cases at least one week of study medication had been taken and at least one post-baseline clinical assessment was completed. Both of these subjects were included in the final analysis using data from the last observation carried forward (LOCF). The single subject not included in the efficacy analysis completed the study, but was found not to have correctly fulfilled the study definition of treatment refractoriness and was excluded. Twenty subjects were allocated to the quetiapine arm and twenty-one to the placebo arm. Student's t-tests were used to determine any baseline differences in the groups for age, gender, number of previous trials of SSRI's, severity of symptoms in relation to OCD, depressive symptoms, and CGI-S. Analysis of variance was undertaken with group and tics as factors. All tests were two-tailed with p-values of less than 0.05 considered significant.

## Results

### Study sample characteristics

For the final analysis, our sample comprised 19 men and 22 women. Baseline characteristics of two treatment groups did not differ with respect to age (years) (quetiapine group 33.8(SD 9.66), placebo group 31.81 (SD 12.14); p = 0.57), gender (p = 0.29), number of previous adequate SRI trials (quetiapine 1.55 (SD 1), placebo 1.62 (SD 1.02) (p = 0.83), baseline severity of OCD (CGI-severity, p = 0.47; YBOCS, p = 0.33), depressive symptoms (p = 0.91), patient-rated disability (p = 0.28) or the presence (n = 11, p = 0.66 and severity (p = 0.87) of co-morbid tics.

### Treatment outcomes

**Figure 1:**
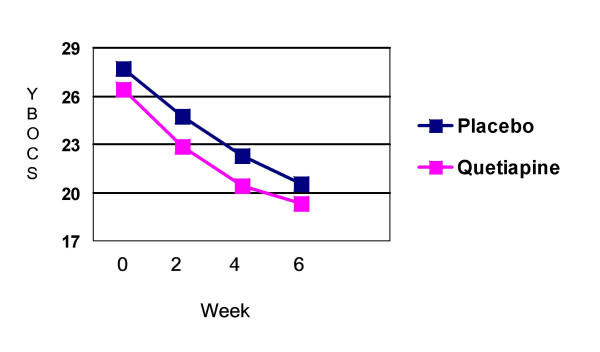
**YBOCS change for treatment groups **Quetiapine and placebo groups improved significantly, without significant between group differences (F = 0.19; p = 0.636)

For the primary outcome measure of severity (YBOCS), quetiapine (p < 0.0001) and placebo (p = 0.001) augmentation of an SRI significantly improved symptoms of OCD. However quetiapine did not demonstrate significant benefit over placebo at the end of the six-week treatment period (F = .19; p = .636) (Figure 1). The mean reduction in YBOCS scores for the combined group was 7.15 points (quetiapine = 7.10; placebo 7.19). Forty percent (n = 8/20) of subjects on quetiapine were classified as responders (YBOCS reduction of >25% from baseline and CGI-improvement score of 1 or 2) while 47.6% (n = 10/21) of subjects on placebo were classified as responders. A higher number of previous SRI trials for the each treatment group did not correlate with the degree of change on the YBOCS or the response status. Table 2 provides details of individual subject SRI doses, baseline clinical severity ratings and response status. Table 3 provides a summary of baseline and change scores for each of the primary and secondary outcome variables.

**Table 2:** Baseline characteristics of treatment groups

Treatment Group		**SRI baseline**	**SRI Dose baseline (mg/day)**	**Previous SRI trials**	**Total YBOCS – Baseline**	**Total YBOCS – Week 6**	**Endpoint dose (mg/day)**	**% CHANGE**	**CGI – Improvement**	**Response status**
**Quetiapine**	1	Paroxetine	60	1	33	29	50	-12.00	3	N/R
	2	Citalopram	60	2	25	23	200	-8.00	4	N/R
	3	Fluvoxamine	300	1	32	27	300	-16.00	3	N/R
	4	Citalopram	70	1	27	25	25 (E/W)*	-7.00	4	N/R
	5	Clomipramine	250	2	22	27	100	23.00	5	N/R
	6	Paroxetine	60	5	35	32	300	-9.00	4	N/R
	7	Fluoxetine	80	3	25	16	300	-36.00	2	R
	8	Sertraline	200	1	18	3	50	-83.00	1	R
	9	Fluoxetine	20	1	21	20	300	-5.00	4	N/R
	10	Fluoxetine	60	1	22	17	300	-23.00	2	N/R
	11	Citalopram	60	1	32	17	300	-47.00	1	R
	12	Fluoxetine	80	1	32	14	300	-56.00	1	R
	13	Fluvoxamine	300	1	30	12	50	-60.00	1	R
	14	Fluoxetine	60	1	25	7	50	-72.00	1	R
	15	Citalopram	60	1	27	24	200	-11.00	4	N/R
	16	Fluvoxamine	300	1	24	12	150	-50.00	2	R
	17	Fluvoxamine	200	2	22	22	50	.00	4	N/R
	18	Fluvoxamine	300	2	25	22	25	-12.00	4	N/R
	19	Fluoxetine	60	2	24	25	25 (E/W)*	4.00	4	N/R
	20	Clomipramine	250	2	27	12	300	-56.00	2	R
										
**Placebo**	1	Fluvoxamine	300	2	32	25	300	-22.00	3	N/R
	2	Fluvoxamine	300	1	30	28	300	-7.00	3	N/R
	3	Fluoxetine	80	2	34	38	300	12.00	4	N/R
	4	Paroxetine	60	1	22	18	300	-18.00	1	N/R
	5	Citalopram	60	1	28	26	300	-7.00	4	N/R
	6	Fluoxetine	60	5	26	24	300	-8.00	3	N/R
	7	Fluoxetine	60	1	23	23	300	.00	4	N/R
	8	Fluoxetine	60	1	27	10	300	-63.00	1	R
	9	Fluoxetine	40	1	32	18	300	-44.00	2	R
	10	Citalopram	60	2	24	23	300	-4.00	4	N/R
	11	Citalopram	60	1	35	23	300	-34.00	2	R
	12	Fluvoxamine	300	3	22	14	300	-36.00	2	R
	13	Citalopram	60	1	28	10	50	-65.00	2	R
	14	Fluoxetine	60	1	28	4	50	-86.00	2	R
	15	Citalopram	60	1	26	19	100	-27.00	2	R
	16	Fluoxetine	60	1	27	12	100	-56.00	1	R
	17	Fluoxetine	20	1	26	18	200	-31.00	2	R
	18	Fluvoxamine	300	2	23	35	200	52.00	6	N/R
	19	Citalopram	60	2	26	9	100	-65.00	2	R
	20	Paroxetine	80	1	32	29	300	-9.00	3	N/R
	21	Fluvoxamine	300	3	31	25	100	-19.00	2	N/R

*E/W = Early withdrawal

**Table 3:** Summary scores (baseline) and change scores for primary and secondary outcome variables.

	Quetiapine	Placebo
YBOCS (baseline)	26.4 (SD4.6)	27.7(SD3.9)
YBOCS (change at week 6)	-7.1(SD7.2)	-7.2(SD8.4)
YBOCS % change	-26.9%	-26%
		
CGI-Severity (baseline)	5.2 (SD0.8)	5.3 (SD0.8)
CGI-Severity (week 6)	4.1 (SD1.4)	4.1(SD1.5)
		
MADRS (baseline)	10.6 (SD 4.8)	10.71 (SD 9.8)
MADRS (change at week 6)	-2.6 (SD 6.5)	-3 (SD 8.3)
		
SDS (baseline)	17.9 (SD 5.3)	19.6(SD4.7)
SDS (change at week 6)	-5.3(SD5.6)	-6.1 (SD4.8)
		
YGTSS (baseline)	24.7 (SD 19.3)	22.6(SD 22.3)
YGTSS (change at week 6)	-4.5(SD 5.1)	-9.4 (SD 14.6)
YGTSS % change	-18.2%	-41.6%

Of the 11 subjects with co-morbid tics, six were randomised to quetiapine. Endpoint data was missing for one subject on quetiapine. Of the remaining 10 subjects, 3 (quetiapine n = 2 (33%); placebo n = 1(20%)) were classified as YBOCS responders. The reduction in the YGTSS did not differ significantly between treatment groups with tics (quetiapine -4.5, placebo -9.4; F = 2.8, p = .46).

Severity ratings for depressive symptoms (MADRS) were low at baseline (mean 10.6, SD 4.8), showed little change over the study period, and at week 6 remained similar for both groups (quetiapine = 8.2, SD 4.8; placebo = 7.7, SD 6.1).

The mean daily dose at week 6 for the quetiapine group was 168.75 mg (SD 120.82) compared to 228.57 mg (SD 99.46) per day for those on placebo. Quetiapine responders (187.5 mg, SD 124.6) did not differ significantly from quetiapine non-responders (156.25 mg, SD 122.1) in their mean daily dose at Week 6 (p = .585). Furthermore, within the quetiapine group, participants receiving ≥ 200 mg/day (10/20 at week 6 demonstrated non-significant differences (F = 6.837, p = .988) and a marginally lower percentage reduction in YBOCS at endpoint (26.7%, SD 20.34) compared to those receiving a dose ≤ 200 mg/day (26.9%, SD 36.24) at endpoint.

### Tolerability

Quetiapine was generally well tolerated and no serious adverse events (SAE's) were reported through the course of the study period. Two patients on quetiapine withdrew from the study due to severe sedation (Week 1 and Week 4) that was judged to be drug related. Otherwise adverse events were in the mild to moderate range and were mostly self-limiting. No subjects on placebo withdrew from the study. Table 4 provides a list of the adverse events and their frequencies in the respective study groups.

**Table 4:** Percentage of subjects for each treatment group reporting adverse events

Adverse event	Quetiapine (%, n)	Placebo (%, n)
Sedation	75% (15)	33.3%(7)
Dry mouth	15% (3)	0
Headache	15% (3)	38% (8)
Fatigue	15% (3)	19% (4)
Irritability	10% (2)	4.7% (1)
Impaired concentration	10% (2)	0
Dizziness	5% (1)	14.3% (3)
Nausea	5% (1)	9.5% (2)
Increased appetite	5% (1)	9.5% (2)
Delayed ejaculation	5% (1)	0
Weight gain	5% (1)	0
Worsening mood	5%(1)	4.7%(1)
Memory difficulties	5%(1)	0
Muscle aches	5%(1)	0
Abdominal tenderness	5%(1)	0
Slurred speech	5%(1)	0

## Discussion

Our findings indicate that both quetiapine and placebo significantly reduced symptoms in subjects with OCD who had failed to respond adequately to 12 weeks of an SSRI and, that the difference between groups was not significant. Similarly in the subgroup with co-morbid tics, no preferential benefit was noted for quetiapine. Interestingly, the high placebo response was similar to that seen in a recent failed controlled trial of olanzapine [25], but stands in contrast to the positive studies in this area in which low placebo response rates were seen when demonstrating efficacy of quetiapine [23], risperidone [21], and olanzapine [24]. It is likely that features of study design or specific study population characteristics may have contributed to this finding and these are discussed below.

First, the duration of a therapeutic trial of an SRI prior to augmentation with an antipsychotic should be of adequate dose and duration. In our study the majority of participants had failed only the single trial of an SRI on which they continued during the study (63.4% mean 1.59). Notably only six weeks of this treatment was required at the maximum tolerated dose. Despite the notion that an optimum trial of pharmacotherapy in OCD is 12 weeks, it may be argued that higher and ultimately effective doses of an SRI had not been maintained for an optimum duration prior to randomization. Given that therapeutic doses of SRIs in OCD are usually on the upper end of the dose range, it seems feasible that the high placebo response rate may reflect a response to SRI's once they had been administered at these higher doses for the additional six weeks of the study. It seems possible that the recent study by Shapira et al [25] may have been impacted by similar factors.

In a second and related point; the number of previous SRI trials in the subgroup receiving quetiapine did not predict a poorer response to treatment. This effect is probably related to the lack of statistical power to detect these differences in a group in which the low number of previous SRI trials was a distinguishing characteristic. Certainly, previous positive studies in this area have used relatively more refractory groups based on the number of previously failed SRI trials. Taken together with the first point above, we suggest that future work in this area should consider longer periods at maximum tolerated doses of SRI's prior to categorisation of subjects as treatment refractory.

Third, the use of a slow up-titration resulted in a relatively low mean daily dose being administered for the majority of the study. For instance, a mean daily (week 6) dose of 168 mg/day in the quetiapine group (median 175 mg) had only been achieved for the final two weeks of the study. These doses are comparably low to those used in the negative single-blind study using low dose quetiapine by Sevincok et al [19]. In contrast the positive study using quetiapine by Denys et al [23] employed a more rapid up-titration and a fixed-dose design. This meant that subjects were exposed to 200 mg daily doses that were generally well tolerated, from the start of week 3. The authors of this study were able to show significant YBOCS differences between groups from the end of week 4. Similarly Mc Dougle et al [21], using risperidone, began treatment on 1 mg per day for one week and permitted weekly 1 mg incremental increases for 6 weeks. They found that by the beginning of week 2, most subjects were on or around the mean daily dose for treatment responders (2.2 mg). Despite the significant improvement in the quetiapine group demonstrated in our study, the apparent lack of benefit of doses higher than 200 mg per day may seem surprising, however, we cannot rule out the possibility that administering these higher doses for an adequate duration would have changed the outcome. In addition it must be noted that in our study the quetiapine group reported high rates of sedation (n = 15, 75%) and a 10% (n = 2) rate of premature withdrawal was experienced. As such it seems likely that a more aggressive up-titration schedule might have resulted in even higher rates of withdrawal. By comparison, rates of sedation were equally high, but did not appear to restrict use of the more rapid up-titration in the study by Denys et al [23]. Certainly evidence of efficacy using lower doses has been demonstrated in studies of 6 and 8 weeks duration [21,23,24], and it seems that therapeutically adequate doses should probably be reached earlier than week 4 in a 6 week study.

Fourth, the impact of repeated clinical assessments and rating of relatively small changes in clinical severity combined with regular dose increases, may conceivably have increased the placebo response rates resulting from increased optimism, a tendency to over-report improvements and belief that higher doses are more likely to be more effective than lower doses. This may be particularly true for the placebo-treated group that were considerably less likely to report sedation as an adverse event and as such were more likely to have their treatment dose increased at each visit. Our results differ, with respect to placebo response, from a considerable literature that suggests a consistently lower placebo response rate in treatment trials in OCD than in other mood and anxiety disorders. While we believe that the reasons (1–3) discussed above probably provide the main reasons for our finding, the impact of repeated assessments and the potential effect thereof cannot be entirely discounted.

## Conclusions

Despite significant improvement in each of the study groups, response to quetiapine augmentation in SRI non-responders, failed to separate from placebo treated subjects at the end of the six week treatment phase. A number of limitations in study design make comparisons with previous studies in this area difficult and probably contributed to our negative findings. Future work in this important clinical area should address these limitations.

## Competing interests

Dr. Stein has received research grants and/or consultancy honoraria from Astrazeneca, Eli-Lilly, GlaxoSmithKline, Lundbeck, Orion, Pfizer, Pharmacia, Roche, Servier, Solvay, Sumitomo, and Wyeth.

## Authors' contributions

PDC drafted manuscript and analysed data, BV protocol design, SS protocol design, JEM drafting of manuscript, statistical design, data acquisition, MVM protocol design, DJS principal investigator, protocol design, drafting of manuscript.

## Pre-publication history

The pre-publication history for this paper can be accessed here:


